# Health Justice and Systems of Care: A Required Longitudinal Course for MD Students

**DOI:** 10.5334/pme.1325

**Published:** 2024-06-21

**Authors:** Ronan Hallowell, Jacob Schreiber, Sonali Saluja, Danica Liberman, Donna Elliott

**Affiliations:** 1 Health Justice and Systems of Care, Department of Medical Education, Keck School of Medicine of the University of Southern California, Los Angeles, California, US; 2 Department of Medical Education, Keck School of Medicine of the University of Southern California, Los Angeles, California, US; 3 Healthcare in Action, Long Beach, California, US; 4 Health Justice and Systems of Care, Department of Emergency Medicine, Department of Population and Public Health Sciences, Keck School of Medicine of the University of Southern California and Children’s Hospital Los Angeles, Los Angeles, California, US; 5 Department of Medical Education and Vice Dean for Medical Education, Keck School of Medicine of the University of Southern California, Los Angeles, California, US

## Abstract

**Problem & Background:**

Medical education has acknowledged the impact of structural societal factors on health, prompting the need for curricula seeking to eliminate health inequities upstream while simultaneously caring for downstream effects of existing inequities. The Keck School of Medicine of USC (KSOM) implemented one such comprehensive curriculum, Health Justice and Systems of Care (HJSC), integrating health systems science, structural competency, and service-learning in a required course spanning the pre-clerkship and clerkship phases with an optional post clerkship elective.

**Approach:**

The HJSC course addresses topics including racism in medicine, health inequities, and health systems science. Using transformative learning theory, it fosters critical consciousness and structural competency. Assessments include case analyses, reflections, team-based learning sessions, and group projects related to social justice in healthcare. The program aims to instill cultural humility and practical application, fostering a holistic approach to medical education that implores physicians to become advocates for health justice.

**Outcomes of the Innovation:**

Feedback from students indicated generally positive perceptions of the curriculum. Students provided overall positive comments about discussions with guest speakers. However, students expressed a desire for more concrete examples of how health inequities can be remedied. Some found small-group activities less engaging. Other challenges included providing students of different readiness levels with tailored experiences and seamlessly integrating HJSC content within basic and clinical sciences courses.

**Critical Reflection:**

Next steps include continuing to integrate content into the science curriculum and clerkships, improving opportunities for meaningful student interactions, and enhancing faculty development to address health justice concerns in clinical settings.

## Background & Need for Innovation

In the 21^st^ century, medical educators have started to acknowledge the significant impact that structural and social factors have on people’s health [1,2]. To ameliorate these historically generated and deeply entrenched structural inequities, physicians, medical students, and other healthcare and policy professionals must learn how these inequities have arisen, how systems continue to perpetuate them, and the steps needed to eliminate them [3,4]. Healthcare professionals must also, collaboratively, learn how to identify gaps in the health system, design solutions, implement changes, and analyze their effectiveness. Over the past 5–10 years, and especially considering the COVID-19 pandemic and the renewed call for racial reckoning, many medical schools, and their accrediting bodies have called for the development of curricula to address these issues [5]. For these reasons, Health Justice and Systems of Care (HJSC) is a required, longitudinal pre-clerkship and clerkship phase course at the Keck School of Medicine of USC (KSOM) that introduces the perspectives and tools of health systems science [6], structural competency [7], and service-learning [8] to inspire students to envision and work toward interventions and policies that will lead to health justice.

Several studies have reported on social justice or social medicine curricula at medical schools (mostly describing elective courses) [9]. However, we have not identified any reports of required longitudinal and comprehensive integrated health justice and health systems science curricula that span both the pre-clerkship and clerkship phases.

## Goal of Innovation

The KSOM Doctor of Medicine Program recently underwent a curriculum renewal that introduced a required longitudinal Health Justice and Systems of Care (HJSC) course. This course comprises didactics in the first three semesters of medical school focused on topics such as racism in medicine, structural determinants of health, health inequities, marginalized populations, and health systems science. During the first two semesters, students also engage in experiential learning with a community-based organization through the service-learning program. During the clerkship phase, students participate in a two-week HJSC intensive seminar that delves deeper into the clinical ramifications of health justice and health systems science topics that includes racism in medicine, advocacy, team-based medicine, high-value care, trauma informed care, disability rights, and gender affirming care. Pursuing a transformative learning [10] framework, the objective is to cultivate cultural humility and apply the learning practically, encouraging a comprehensive approach to medical training that urges doctors to champion health justice.

## Steps Taken for Development and Implementation of Innovation

In 2018 senior leadership launched the planning phase of a curriculum renewal initiative. During that phase, subject matter experts and medical educators were tasked with envisioning how best to deliver health systems science curriculum in a new format to update the first iteration launched in 2017. Through a series of convenings of faculty, students, and community members, as well as curriculum committee meetings, and the work of a student led anti-racism taskforce in 2020–2021, relevant parties determined that our approach to health systems science would be best framed and organized to reflect the school’s commitment to health justice. Our guiding principle is that if health systems science education is to be successful it must lead to health equity.

The course development process also included an American Medical Association (AMA) grant-funded pilot course titled *Advocacy for Health Justice* [3] that was delivered in spring 2021. The pilot course, that was co-taught by leaders from local community-based organizations and university faculty, was designed with a group of fourth year medical students with a keen interest in health justice issues, some of whom had recently completed a Master of Public Health degree and wanted to translate their capstone work on community engagement and advocacy into a fourth-year elective. In addition to student feedback from evaluations, students contributed to the development of the HJSC course by serving as representatives on curriculum committees, a bi-annual advisory group, and outreach to course directors when they had feedback to share. On several occasions students helped design and lead sessions on topics such as residential segregation, serving deaf patients, and medical student advocacy. The 24 community organizations that participate in the service-learning program provide their input in the form of annual surveys and through regular communication with the director of the service-learning program. Some organizations, such as Homeboy Industries who provide formally incarcerated gang-members tattoo removal and health education services, have had long-standing relationships with the medical school while others, such as Seeds of Hope– a food justice organization– are recruited by the director of service learning who is active in cultivating relationships in the community.

This development process led to our current, longitudinal *Health Justice and Systems of Care (HJSC)* curriculum that launched in fall 2021 and is required for all students in the pre-clerkship and clerkship phases, with an optional senior seminar in *Advocacy and Policy* in the post-clerkship phase. Commitment from the senior leadership of the medical school has provided space in the overall curriculum and resources needed to staff the program.

### Instructional Approach Grounded in Transformative Learning Theory

Recognizing that persistent health inequities in the United States and a dysfunctional and costly health care system that perpetuates inequality needs serious reform, the HJSC course uses transformative learning theory [10] as a framework for teaching critical consciousness, structural competency, and health systems science. Students are guided through a process of reflection, questioning, and personal growth in and outside of the classroom throughout the course. In the classroom, ‘disorienting dilemmas’ are intentionally created for students to confront their pre-existing assumptions about social structures, healthcare, and their roles within these systems. Service-learning fosters a deep connection between the concepts introduced in the pre-clerkship sessions and real-world practical application of those concepts at local health, education and community-based organizations. This approach encourages open dialogue and the exploration of diverse perspectives to encourage students to develop a critical consciousness that Freire describes as “learning to perceive social, political, and economic contradictions…so that individuals can take action against the oppressive elements of reality [11].”

### Program Overview

In the pre-clerkship phase, HJSC is delivered as a three-semester, 22 session course that occurs approximately every other week. Expert guest speakers or panels usually address the students during the first hour of each session. Afterwards, students spend another hour engaging in small group activities led by faculty facilitators who are sociologists, anthropologists, physicians, and educators with expertise in social medicine. This structure is interspersed by team-based learning (TBL) sessions throughout the course. In the first year, students also complete 12-hours of service learning as part of the course, which allows for challenging assumptions and learning first-hand from members of the community surrounding the KSOM campus.

**Figure 1:**
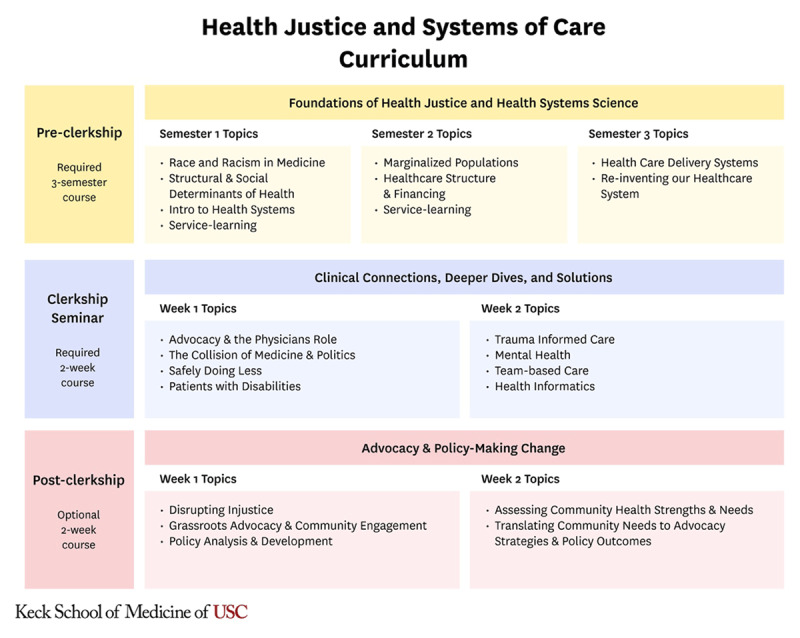
Health Justice and Systems of Care Course Overview.

During the clerkship phase there are HJSC related sessions in various clerkships. For example, the internal medicine clerkship students reflect on how structural inequities impact the health of one of their patients. This reflection prompts them to question and critically examine their own preconceived notions and the systemic biases that exist within healthcare systems. This aligns with transformative learning, where critical reflection acts as a catalyst for personal change. To allow students to consolidate information from the pre-clerkship course and their clinical experiences they participate in a required 2-week seminar featuring speakers, panels, small group activities, and a group project. Transformative learning emphasizes the importance of experience in learning, proposing that the way learners interpret and reinterpret their experience is central to making meaning and hence learning. The seminar provides space for students to take a step back and reinterpret their clinical experience using the health justice frameworks introduced in the pre-clerkship and encountered in action on the wards. The optional post-clerkship senior seminar is a two-week full time intensive in which students learn from community-based organizations and subject matter experts about advocacy issues. By applying a transformative learning approach to advocacy, students link theoretical knowledge with practical applications and are not only informed but are also encouraged to take action that can help transform the status quo. Figure 1 depicts the HJSC course.

The course begins in the first semester of the pre-clerkship phase by addressing core health justice issues in the United States including: the historical roots of contemporary inequities, how structural determinants of health drive poor health outcomes for marginalized populations and the need for the medical profession to practice cultural humility and sincere community engagement. Towards the end of the first semester and into the beginning of the second semester, students are introduced to issues in health systems science pertaining to how the U.S. health system is structured and financed so students can begin to understand how the system works and explore different proposals for improving it. In the mid-part of the second semester, a series of sessions on topics related to marginalized populations such as LGBTQ+ health, prison and jail health, homelessness, and immigrant health give students an opportunity to examine how structural drivers of health manifest in particular ways for specific populations. During the first two semesters students engage in service-learning in the local community to see how issues discussed in class play out in real life and to develop relationship building skills rooted in cultural humility. During the third semester we return to an emphasis on health systems science issues such as population health, international health systems, and equity in access to medications as students work on a culminating group project aimed at envisioning health system solutions capable of fostering health equity.

During the clerkship seminar students are asked to reflect on their clinical experiences and make connections between what they’ve encountered on the wards with the topics addressed in the course. As in the pre-clerkship phase, health justice focused issues such as medicine and politics and further exploration of marginalized populations are interspersed with health systems science topics such as value-based care and health informatics. The health systems science topics are approached in ways that help students think of actionable strategies to improve health systems in the U.S.

## Outcomes of Innovation

Students are assessed through case/scenario analyses, reflections, TBL sessions, and a culminating group project at the end of each phase. The small group faculty facilitators assess the assignments using rubrics. The TBL sessions also include multiple choice quizzes. In the semester one scenario analysis, students examine how race-based medicine, rooted in the fallacious assumption that race is a biological fact rather than a socio-political construction, can manifest at different levels of the health system from the interpersonal to the institutional. Subsequently, students identify ways in which to apply race-conscious medicine that recognizes racism has deleterious health consequences due to chronic stress, while eschewing the notion that race itself is a risk-factor for disease. In the second semester, students analyze a complex case from an intersectional lens. The assignment requires critically transferring lessons learned from studying the concerns of several marginalized populations to articulate a structurally competent and culturally responsive care plan.

Students also complete several written reflections across the three semesters of the pre-clerkship. These assignments challenge students to 1) consider how they will act with cultural humility as they work with people from diverse backgrounds, 2) reflect on how to apply lessons learned from at least one specific session to their future practice, 3) advocate for change in the health system and society more broadly, and 4) appraise their service-learning experiences for connections to themes explored throughout course.

At the end of the third semester, students complete a group project examining and proposing a solution to ameliorate one specific social justice issue in healthcare. During the clerkship, students also complete reflections, case-based analyses, and a group project that connects what they have learned in the clinical environment to the topics they previously explored in the course.

Faculty who reviewed assignments indicated that most students exhibited satisfactory critical and reflective thinking skills at a level consistent with the course objectives. For some of the TBL sessions, a few students failed the individual quiz based on the required pre-work. These students had to complete a written make-up assignment. Outside of the traditional assessments described above, we attempted to measure the development of critical consciousness – the ability to recognize systems of oppression, act against them and transform social and material reality [11]—using a pre-post short Critical Consciousness Scale [12] with our first cohort of students. They completed the scale before the start of the first semester of the course and again at the end of the pre-clerkship phase. We did not find any statistically significant results indicating an increase in critical consciousness. This may be in part because the scale had shown evidence of validity with results from a population of high school students which may not translate to medical students. In the future, we might consider modifying this scale to account for specific factors related to the medical student population. However, this was beyond the scope of our capacity at this juncture of the course’s evolution.

### Analysis of evaluation data

Students provided feedback about each session of HJSC individually and each semester overall as a regular component of the course and faculty evaluation. Students rated their perceptions of the overall educational value of a semester of material on the following scale 1) Poor, 2) Fair, 3) Good, 4) Very Good, 5) Excellent. The results of this satisfaction data are presented here to describe the students’ responses to the implementation of HJSC and promote the discussion of the way the HJSC curriculum was continuously modified to respond to challenges integrating the material into an undergraduate medical education program. The average ratings for each semester of HJSC that the classes of 2025 and 2026 have completed thus far appear in Table 1.

**Table 1:** Student Ratings of HJSC Content by Semester.


	CLASS OF 2025			CLASS OF 2026		
	
	n	M	SD	n	M	SD

Semester 1	62	3.18	0.99	143	3.51	1.04

Semester 2	119	4.01	0.96	117	3.46	1.02

Semester 3	143	3.51	1.04	144	3.44	1.06

Clerkship Seminar	131	3.34	1.14	Spring 2025		


In addition to the ratings, students were prompted to describe what they specifically liked about the course and anything they felt could be improved. For the analysis of this qualitative feedback, the second author employed a thematic analysis approach [13] to illuminate key points within the data. Throughout the course, most students praised the large group discussions with guest speakers and expert panels in their comments. They described those talks as engaging and thought-provoking. However, some students critiqued the small-group activities that followed the speaker sessions and indicated their engagement with their peers was often superficial.

Many comments echoed that the large group activities offered greater educational benefit than discussing the issues and working through assignments in the small groups. Students often expressed that they did not have the context at this point in their training to consider solutions to major sociological health issues. For example, one student from the class of 2025 wrote:

I felt that the concepts in HJSC were quite lofty and difficult to wrap our heads around … letting the panelists speak and debate more would have given us more insight into the topic rather than us going back to the [small groups] to do the activity.

While this comment indicated the student was struggling with the material and desired more input from expert sources, other students critiqued the course for being too introductory. The disagreement in the comments about the depth of the course was revealing of the difficulties of meeting students at various points of readiness to engage with the material. Indeed, the preference students showed for the more passive large group portions of the sessions may be indicative of a reluctance to personally confront challenging issues. The struggle was exacerbated by contradictory information students learned outside of the HJSC course. For example, one student from the class of 2026 wrote:

We often learn about something in HJSC and how doctors sometimes mistreat particular patients based on race/housing status/class/drug use and then in [other courses/settings] we see that exact poor treatment replicated.

Based on student feedback, there are successes and areas for improvement to report about the first iteration of HJSC. Overall, students enjoyed hearing from experts in the field and most of them see the value of learning about this material.

## Critical Reflection

Specific challenges arise when educating medical students about health justice and systems of care, necessitating additional thought. Creating environments where students of differing knowledge levels about social issues can interact effectively is challenging. Although we have not devised a satisfactory way to systematically determine levels of prior knowledge and operationalize them in small groups, we halved the student-to-facilitator ratio to 12–1 for the next cohort, which we hypothesize will allow for more intimate discussion, promote facilitators’ assessment of students’ prior knowledge, and facilitate effective engagement across those knowledge levels. Additionally, integrating the course seamlessly into the broader curriculum to ensure that health justice is a pervasive element throughout their educational journey is an on-going task. Considering the amount of diverse material involved with health justice issues, health systems science, and the complex social nature of many of the skills that contribute to concepts like structural competency, it has been difficult to define clear outcome measures that are manageable to assess.

As described in the outcomes section, there were no statistically significant changes in the first cohort of students’ scores on our pre-post implementation of the critical consciousness scale. However, this scale was designed for adolescents and may not specifically reflect the ways medical students enact their critical consciousness. Adaptation of the scale for the medical student population was outside of the scope of this project but remains a limitation that we seek to address in future work. We did not find other reliable scales that appeared to measure the curricular outcomes. We did consider a “social foundations of health evaluation instrument” that Metzl and Petty [14] developed to assess structural competency in pre-health undergraduate students at Vanderbilt University. However, the instrument required an excessive amount of qualitative data analysis outside of regular faculty assessment duties and would not be feasible to implement as a regular tool of assessment.

We are also exploring ways of assessing HJSC competencies through workplace-based assessments with students in clinical environments. We are undertaking this work through an AMA “ChangeMedEd” grant that includes seven other medical schools across the U.S. Our consortium of schools is developing a workplace-based assessment tool for clerkship and sub-internship students using elements of the entrustable professional activities framework [15] that incorporates health systems science competencies such as systems thinking and the inclusion of the structural and social determinants of health in patient care plan development. Work with this team has indicated that many schools that are implementing health systems science and health justice curricula are facing challenges of how to assess students and evaluate programs due to the complex nature of the material that does not easily lend itself to simple assessment approaches. More robust assessment modalities such as workplace-based and written assessments require significant faculty time and analyzing them to determine if program outcomes are achieved requires another level of qualitative data analysis that can be hard for course directors to complete in an efficient manner.

Ratings and comments from KSOM student satisfaction surveys described in the outcomes section have been important for identifying specific issues and challenges relevant to the integration of HJSC into the biomedical curriculum in the early stages of implementation. For example, Table 1 noted the first semester HJSC exhibited the lowest average rating. Feedback from students in evaluations and in discussions with the course directors indicated they felt a need for more concrete examples of positive changes made in specific health justice issues to serve as a roadmap for how to think about relevant ways for physicians to ameliorate problems raised throughout the course. Subsequently, a concerted effort has been made to provide as many examples as possible of constructive ways that health professionals may engage with policy makers and concerned citizens to galvanize positive change. As demonstrated in the outcomes section, satisfaction with Semester 1 scores increased slightly following the modifications.

Key next steps are to continue to integrate HJSC content more seamlessly into the basic and clinical science curriculum and within the clerkships, not just the two-week HJSC clerkship-phase seminar. We are also working closely with our local experts to improve opportunities for more meaningful student interaction in the small group activity portion of the course and in other areas of the curriculum. Improving faculty development across the clinical enterprise so that attending-physicians have the skills to be good examples to students in their handling of health justice concerns is another key priority. Although the course has been well received and supported at our institution, we recognize that there has been significant backlash against this type of work in some regions of the United States that is complicating an already complex landscape and erecting additional barriers to addressing long-standing inequities. We are working with other health professions schools and professional organizations to build a stronger national coalition to advocate for substantive change. Individuals and institutions committed to this work will need to adopt a long-term strategy and continue to dedicate resources to achieve health justice in the coming years and decades.

## Previous presentations

Portions of this work have been described nationally in forms of panel and oral presentations at the following conferences: American Medical Association Health Systems Science Summit, December 6, 2022. Chicago, Illinois; AAMC Western Group on Educational Affairs Annual Conference, April 15, 2023, University of Hawaii, Honolulu, Hawaii.
